# Functional Interaction between Type III-Secreted Protein IncA of *Chlamydophila psittaci* and Human G3BP1

**DOI:** 10.1371/journal.pone.0016692

**Published:** 2011-01-31

**Authors:** Nicole Borth, Katrin Litsche, Claudia Franke, Konrad Sachse, Hans Peter Saluz, Frank Hänel

**Affiliations:** 1 Department of Cell and Molecular Biology, Leibniz Institute for Natural Product Research and Infection Biology (Hans Knoell Institute), Jena, Germany; 2 Friedrich-Schiller-University, Jena, Germany; 3 Friedrich-Loeffler-Institut (Federal Research Institute for Animal Health), Institute of Molecular Pathogenesis, Jena, Germany; University Freiburg, Germany

## Abstract

*Chlamydophila (Cp.) psittaci*, the causative agent of psittacosis in birds and humans, is the most important zoonotic pathogen of the family *Chlamydiaceae*. These obligate intracellular bacteria are distinguished by a unique biphasic developmental cycle, which includes proliferation in a membrane-bound compartment termed inclusion. All *Chlamydiaceae spp.* possess a coding capacity for core components of a Type III secretion apparatus, which mediates specific delivery of anti-host effector proteins either into the chlamydial inclusion membrane or into the cytoplasm of target eukaryotic cells. Here we describe the interaction between Type III-secreted protein IncA of *Cp. psittaci* and host protein G3BP1 in a yeast two-hybrid system. In GST-pull down and co-immunoprecipitation experiments both *in vitro* and *in vivo* interaction between full-length IncA and G3BP1 were shown. Using fluorescence microscopy, the localization of G3BP1 near the inclusion membrane of *Cp. psittaci*-infected Hep-2 cells was demonstrated. Notably, infection of Hep-2 cells with *Cp. psittaci* and overexpression of IncA in HEK293 cells led to a decrease in c-Myc protein concentration. This effect could be ascribed to the interaction between IncA and G3BP1 since overexpression of an IncA mutant construct disabled to interact with G3BP1 failed to reduce c-Myc concentration. We hypothesize that lowering the host cell c-Myc protein concentration may be part of a strategy employed by *Cp. psittaci* to avoid apoptosis and scale down host cell proliferation.

## Introduction

The avian and human pathogen *Cp. psittaci* is the causative agent of psittacosis and represents the most important animal chlamydiosis of zoonotic character [1]. In addition, recent surveys showed that *Cp. psittaci* can also be found in non-avian domestic animals and wildlife [2], [3]. All members of the family *Chlamydiaceae* are obligate intracellular parasites that develop in a host cell within an inclusion, i.e. a membrane-bound compartment that does not fuse with lysosomes [4]. The membrane of the inclusion is initially formed by invagination of the plasma membrane and pinching off of a vesicle containing the infectious form of the bacterium, the elementary body (EB). Thereafter, EBs differentiate into non-infectious but metabolically active reticulate bodies (RB), which proliferate within the expanding inclusion, giving rise to 1000 or more progeny per host cell. The developmental cycle ends after 2–3 days depending on the strain, when RBs transform back into EBs and are released into the extracellular medium [5]. During this unique biphasic developmental cycle replicating bacteria acquire energy and biosynthetic precursors from the infected cell. Furthermore, chlamydiae modulate cellular functions such as apoptotic programs and immune response [6], [7]. Studies on inhibitors of bacterial protein synthesis suggest that modulation of the host cell functions requires the activity of chlamydial proteins. All *Chlamydiaceae ssp.* possess genes encoding core components of a Type III Secretion (TTS) apparatus [8], a protein transport system used by Gram-negative bacteria to translocate proteins into the cytoplasm of the host cell. Therefore, it is commonly accepted that chlamydial effector proteins are targeted by the TTS to the inclusion membrane. The first set of chlamydial effector proteins identified was a family of integral inclusion membrane (Inc) proteins that share one remarkable feature, i.e. they possess a very large (50–80 amino acids) bilobed hydrophobic domain, a secondary structure motif predictive of protein localization to the chlamydial inclusion membrane [9], [10]. The first member of the family of Inc proteins identified, IncA, is the one that has attracted most of the attention. First cloned in *Cp. caviae* it has known homologs in *Chlamydia* (*C.*) *trachomatis*, *Cp. pneumoniae, Cp. felis, C. muridarum, Cp. abortus* and *Cp. psittaci.* The level of sequence similarity among the homologs is low, and antibodies against IncA do not cross-react with other chlamydial species. Furthermore, in all IncA proteins identified so far, SNARE (soluble N-ethylmaleimide-sensitive factor attachment protein receptor) – like motifs were identified [5]. These motifs allow interactions with several host SNARE proteins, which are essential for membrane fusion [11], [12]. In addition to the bilobed hydrophobic domain Inc proteins, such as IncA and IncG, harbor domains exposed to the cytoplasmic side of chlamydial inclusion where they mediate interactions with eukaryotic host proteins such as Rab GTPases [13], [14], 14-3-3β protein [15], and Act1 [16]. Thus, Inc proteins are probably central regulators of pathogen-host interactions.

Ras-GTPase activating protein SH3 domain binding protein 1 (G3BP1) was initially identified as an ubiquitously expressed cytosolic 68 kDa protein that co-immunoprecipitates with Ras-GTPase-activating protein (GAP). The G3BP1 cDNA revealed that G3BP1 is a 466-amino-acid protein that shares several features with heterogeneous nuclear RNA-binding proteins, including RNA recognition motifs (RRM) RNP1 and RNP2, an RG-rich domain and acidic sequences [17]. G3BP1 colocalizes and physically interacts with GAP at the plasma membrane of serum-stimulated but not quiescent Chinese hamster lung fibroblasts. In quiescent cells, G3BP1 was hyperphosphorylated on serine residues and harbors a phosphorylation-dependent RNase activity which specifically cleaves the 3′-untranslated region of human *c-myc* mRNA [18]. In addition of its role in Ras-GAP signalling and its function as a phosphorylation-dependent RNase several other putative biological activities of G3BP1 were suggested, i.e. involvement in NFκB and IκB nucleo-cytoplasmic equilibrium, interactions with ubiquitin-specific proteases and participation in stress-granule formation (reviewed in [19]). Moreover, the G3Bp family of proteins is evolutionarily conserved throughout eukaryota [19].

In this study, we describe the interaction between the Type III-secreted protein IncA of *Cp. psittaci* [20] and the host protein G3BP1 in a yeast two-hybrid system. While there is a growing list of publications dealing with interactions between Incs from *C. trachomatis* and *Cp. pneumoniae,* this is, to our knowledge, the first example of a documented interaction between an Inc protein from a zoonotic chlamydia and a host protein. In GST-pull down and co-immunoprecipitation experiments, both *in vitro* and *in vivo* interaction between full-length IncA and G3BP1 could be shown. Using fluorescence microscopy the localization of G3BP1 near the inclusion membrane of *Cp. psittaci*-infected Hep-2 cells was demonstrated. Finally, infection of Hep-2 cells with *Cp. psittaci* and overexpression of IncA in HEK293 cells led to a decrease in c-Myc protein concentration, but not at mRNA level. This effect could be ascribed to the interaction between IncA and G3BP1 since overexpression of an IncA mutant construct disabled to interact with G3BP1 did not cause a decrease in c-Myc concentration. Additionally, siRNA mediated knock-down of G3BP1 in Hep-2 cells had the same influence on c-Myc protein level as overexpression of IncA.

## Results

### IncA of *Cp. psittaci* and the host protein G3BP1 interact in a yeast two-hybrid system

**Figure 1:**
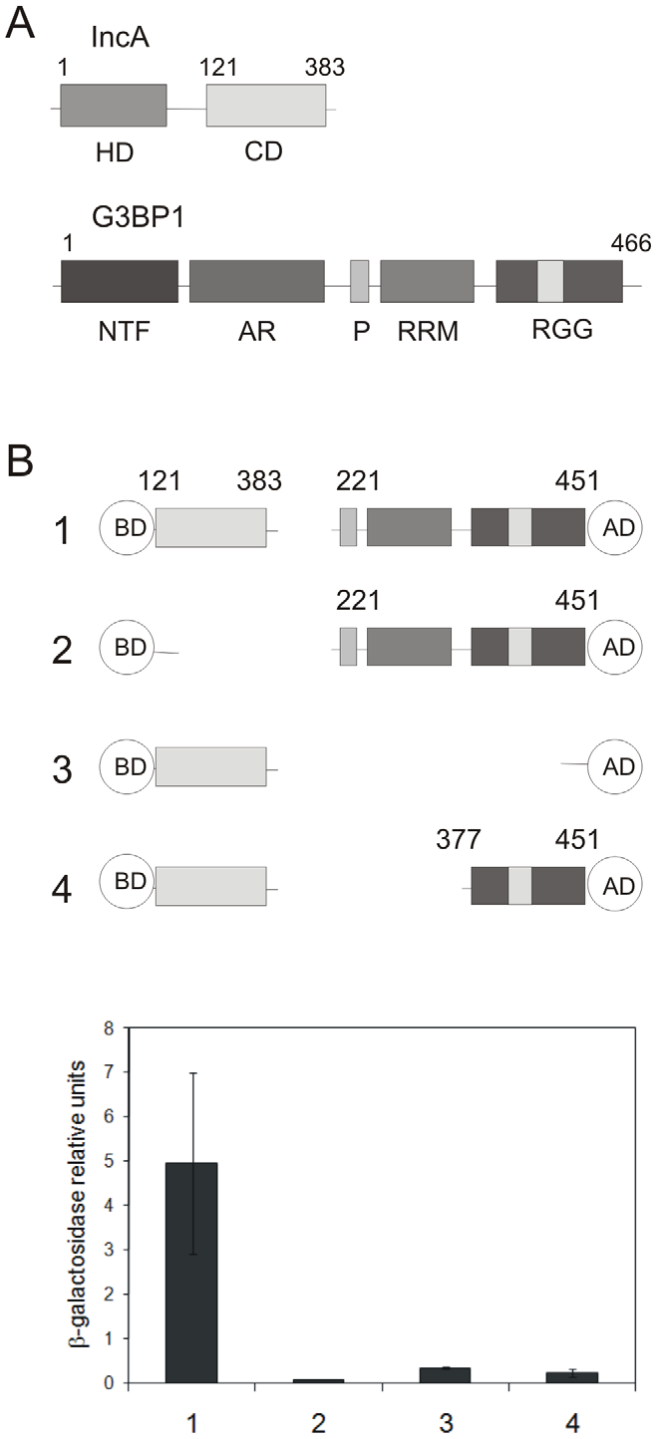
IncA of *Cp. psittaci* interacts with human G3BP1. (A) Schematic structures of IncA of *Cp. psittaci* and human G3BP1. Abbreviations: HD, hydrophobic domain; CD, cytoplasmic domain; NTF2, Nuclear Transport Factor 2-like domain; AR, acid rich region; P, proline rich motif; RRM, RNA recognition motif; RGG, arginine-glycine rich box. (B) Specific interaction of the IncA-CD fused to GAL4 DNA-binding domain (BD) with C-terminal part (aa 221-451) of G3BP1 fused to GAL4 activation domain (AD) in yeast. Interactions were quantitatively assessed by β-galactosidase liquid culture activity assays. β-galactosidase values represent the mean numbers and standard deviations of three independent experiments.

To gain insights into pathogenicity mechanisms of zoonotic *Chlamydiaceae*, we chose the cytoplasmic domain (aa 121-383) of the inclusion membrane protein IncA from *Cp. psittaci* DC15 (Fig. 1A) as a bait in a yeast two-hybrid screen for specific interactions with target eukaryotic proteins. The localization of IncA from *Cp. psittaci* in the inclusion membrane of infected cells was shown very recently by fluorescence microscopy and immuno electron microscopy [20]. Expression of the cytoplasmic domain of IncA from *Cp. psittaci* fused to the GAL4 DNA-Binding domain (BD) in pGBKT7 (CLONTECH) in *Sacharomyces (S.) cerevisiae* Y190 (CLONTECH) did not activate any of the reporter genes (*HIS3*, *lacZ*) (Fig. 1B). Therefore this strain was mated with *S. cerevisiae* Y187 expressing the HeLa cDNA MATCHMAKER 3 library in the activation domain (AD) vector pGADT7-Rec (CLONTECH). HeLa cDNA-expressing plasmids from clones growing on high stringency medium were isolated and retransformed into competent yeast expressing BD-IncA (aa 121-383). In the case of positive retransformation HeLa cDNA-expressing plasmids were recovered from these strains, propagated in *Echerichia (E.) coli* and sequenced to identify coding sequences. One isolate contained in frame coding sequence for the C-terminal part (aa 221-451) of human G3BP1 (Fig. 1A). To verify the validity of the interaction *S. cerevisiae* Y190 expressing pGBKT7-IncA (aa 121-383) or only the GAL4 BD were co-transformed with the G3BP1 HeLa-library plasmid and the interaction was quantitatively assessed by β-galactosidase (β-gal) activity assays (Fig. 1B). Compared with the IncA-BD and pGADT7 (column 3) and pGBKT7 and AD-G3BP1 (aa 221-451) (column 2) vector controls, β-gal activity was significantly induced in *S. cerevisiae* Y190 expressing pGBKT7-IncA (aa 121-383) and AD-G3BP1 (aa 221-451) (column 1). The G3BP1 HeLa-library fragment encludes the characteristic RNA recognition motifs of G3BP1 (Fig. 1A). To further narrow down the interaction surface between IncA and G3BP1 we deleted the RRM resulting in an AD-G3BP1 (aa 377-451) construct (Fig. 1B). As shown in Figure 1B, column 4, deletion of the RRM led to the complete loss of interaction with IncA (aa 121-383) in the yeast two-hybrid system. Taken together, these data indicate that the cytoplasmic domain of *Cp. psittaci* IncA specifically interacts with the C-terminal part of human G3BP1 in the yeast two-hybrid system.

### Full-length proteins IncA of *Cp. psittaci* and human G3BP1 interact *in vitro* and *in vivo* in HEK293 cells

**Figure 2 pone-0016692-g002:**
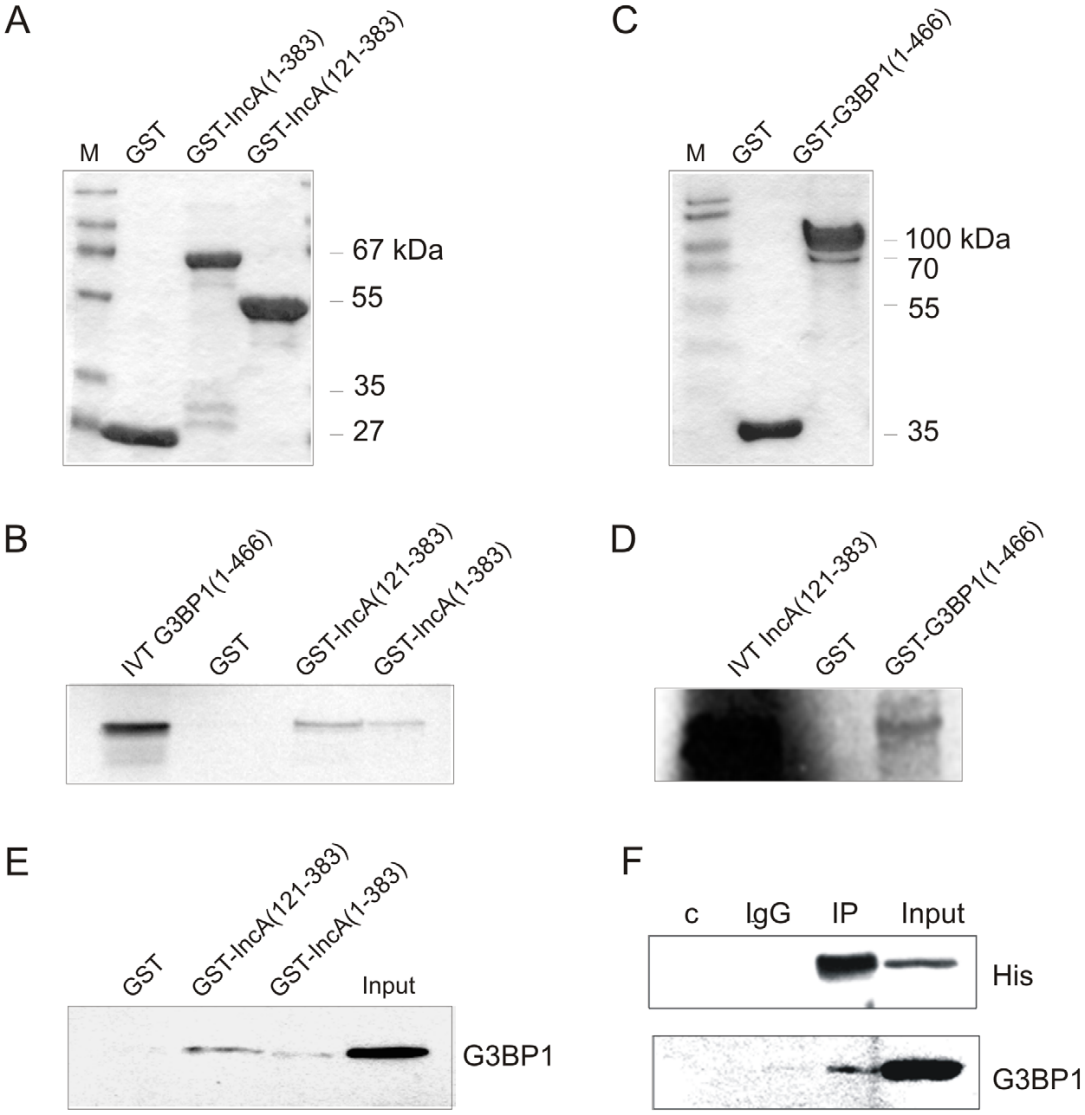
Full-length IncA of *Cp. psittaci* and G3BP1 proteins interact *in vitro* and *in vivo*. (A) Purified GST, GST-IncA (aa 1-338) and GST-IncA (aa 121-383) or (C) GST and GST-G3BP1 (aa1-466) stained with Coomassie. (B) Results of GST pull-down experiments performed with IncA full-length and truncation clone fused to GST and [^35^S]-methionine-labeled full-length G3BP1 or (E) with Hep-2 cell lysate instead of labeled G3BP1. (D) Results of GST pull-down experiment performed with full-length G3BP1 fused to GST and [^35^S]-methionine-labeled IncA (aa 121-383). GST served as control. IVT: 10 µl of the *in vitro* translation product. Input: 25% (75 µg protein) of Hep-2 cell lysate used for the interaction assay. (F) Whole cell lysate of HEK293 cells transfected with a His-tagged codon-adapted IncA mammalian expression construct was subjected to co-immunoprecipitation experiments using anti-His antibody. Co-immunoprecipitated endogenous G3BP1 was detected with a mouse anti-G3BP1 monoclonal antibody. Controls were beads alone (c) or pre-immune serum (lgG). Input: 10% (100 µg) of the protein used for immunoprecipitation.

Next we performed GST pull-down assays to examine whether IncA and G3BP1 directly interact *in vitro*. Glutathione Sepharose beads bound to GST-tagged full-length IncA, GST-IncA (aa 121-383) or GST alone (Fig. 2A) were incubated with [^35^S]methionine-labeled full-length G3BP1, processed as described in Materials and Methods section and analyzed by autoradiography. As shown in Figure 2B, full-length G3BP1 bound specifically to both the GST-IncA (aa 121-383) and the GST-IncA full-length matrix, but no binding was detected using GST as a control, suggesting a direct physical interaction between IncA of *Cp. psittaci* and human G3BP1. In the reversed experiment IncA (aa 121-383) bound specifically to the GST-G3BP1 (aa 1-466) matrix (Fig. 2C and D). In a second approach, we performed a GST pull-down experiment with Hep-2 cell lysates instead of [^35^S]methionine-labeled full-length G3BP1. The associated proteins were pelleted by centrifugation, washed and analyzed for full-length G3BP1 by Western blot analysis (Fig. 2E). Endogenous G3BP1 from Hep-2 cell lysate exclusively bound to GST-IncA (aa 121-383) and GST-IncA and did not interact with GST alone. Finally, in order to show that IncA and G3BP1 also interact *in vivo*, HEK293 cells were transfected with a His-tagged IncA mammalian expression construct. To get a stronger expression of the heterologous protein the codon usage of the IncA gene of *Cp. psittaci* was adapted to human cells. As shown in Figure 2E we were able to co-immunoprecipitate endogenous G3BP1 with His-tagged IncA using an anti-His antibody. No binding was obtained using pre-immune serum or Protein G-Sepharose beads only as negative controls. Consequently, our interaction studies showed that IncA and G3BP1 interact *in vitro* and *in vivo*. The cytoplasmic domain of IncA was proved sufficient to bind the full-length G3BP1.

### G3BP1 is accumulated around the chlamydial inclusion in *Cp. psittaci*-infected Hep-2 cells

**Figure 3:**
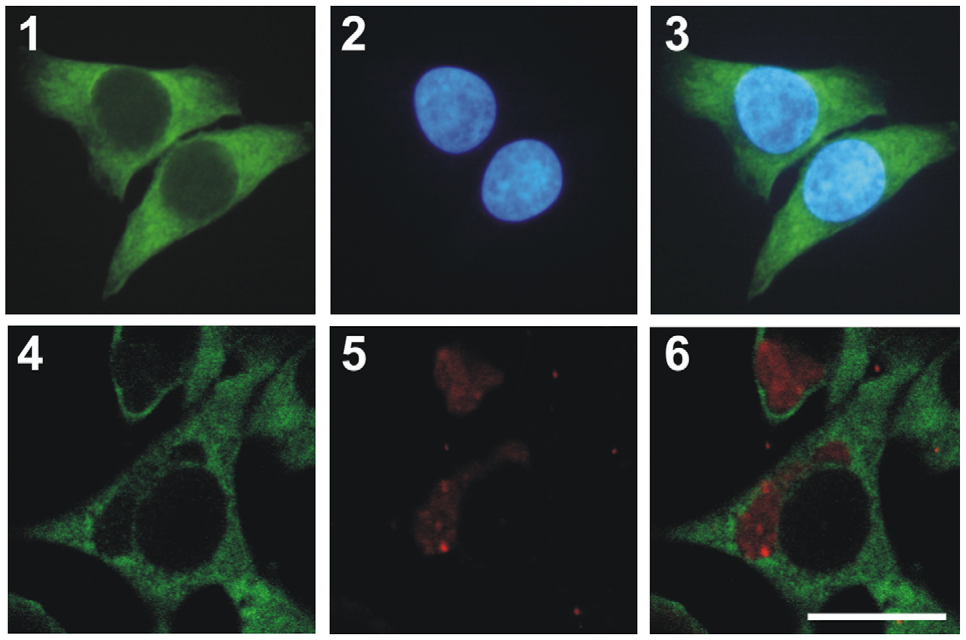
G3BP1 is accumulated around the chlamydial inclusion in *Cp. psittaci* infected Hep-2 cells. Hep-2 cells were infected with *Cp. psittaci* at a MOI of 3, and at 48 h postinfection the cells were fixed and stained with anti-chlamydial LPS (red), anti-G3BP1 (green) antibodies and DAPI (blue, nuclei), and viewed under a fluorescence (1–3) or a confocal laser-scanning microscope (4–6). Non-infected cells served as control (1–3). The anti-G3BP1 (1 and 4) and anti-LPS (5) or DAPI (2) signals were merged (3 and 6). A fraction of endogenous G3BP1 is concentrated around chlamydial inclusions (6). Bar = 20 µm.

As IncA of *Cp. psittaci* is localized on the membrane of the chlamydial inclusion [20], interaction with G3BP1 should lead to accumulation of G3BP1 in the vicinity of the inclusion. To test this assumption, we performed immunolocalization studies of endogenous G3BP1 in *Cp. psittaci*-infected and non-infected Hep-2 cells using polyclonal rabbit anti-G3BP1 antibody (Fig. 3). In non-infected Hep-2 cells, G3BP1 showed a uniformly cytoplasmic localization (Fig. 3, 1–3). In *Cp. psittaci*-infected Hep-2 cells, a fraction of G3BP1 was found to be concentrated around the chlamydial inclusion as shown in Fig. 3 (4–6, inclusion in red). An alternative approach, the transfection of infected Hep-2 cells with a DsRed-G3BP1 expression vector, gave disappointing results because overexpression of G3BP1 induced assembly of stress granules [21], [22] which prevented any further study (data not shown). In summary, the immunolocalization data show an accumulation of G3BP1 around chlamydial inclusions which is due if IncA and G3BP1 interact.

### Infection of Hep-2 cells with *Cp. psittaci* leads to a decrease in c-Myc protein concentration, but does not affect *c-myc* mRNA concentration

**Figure 4:**
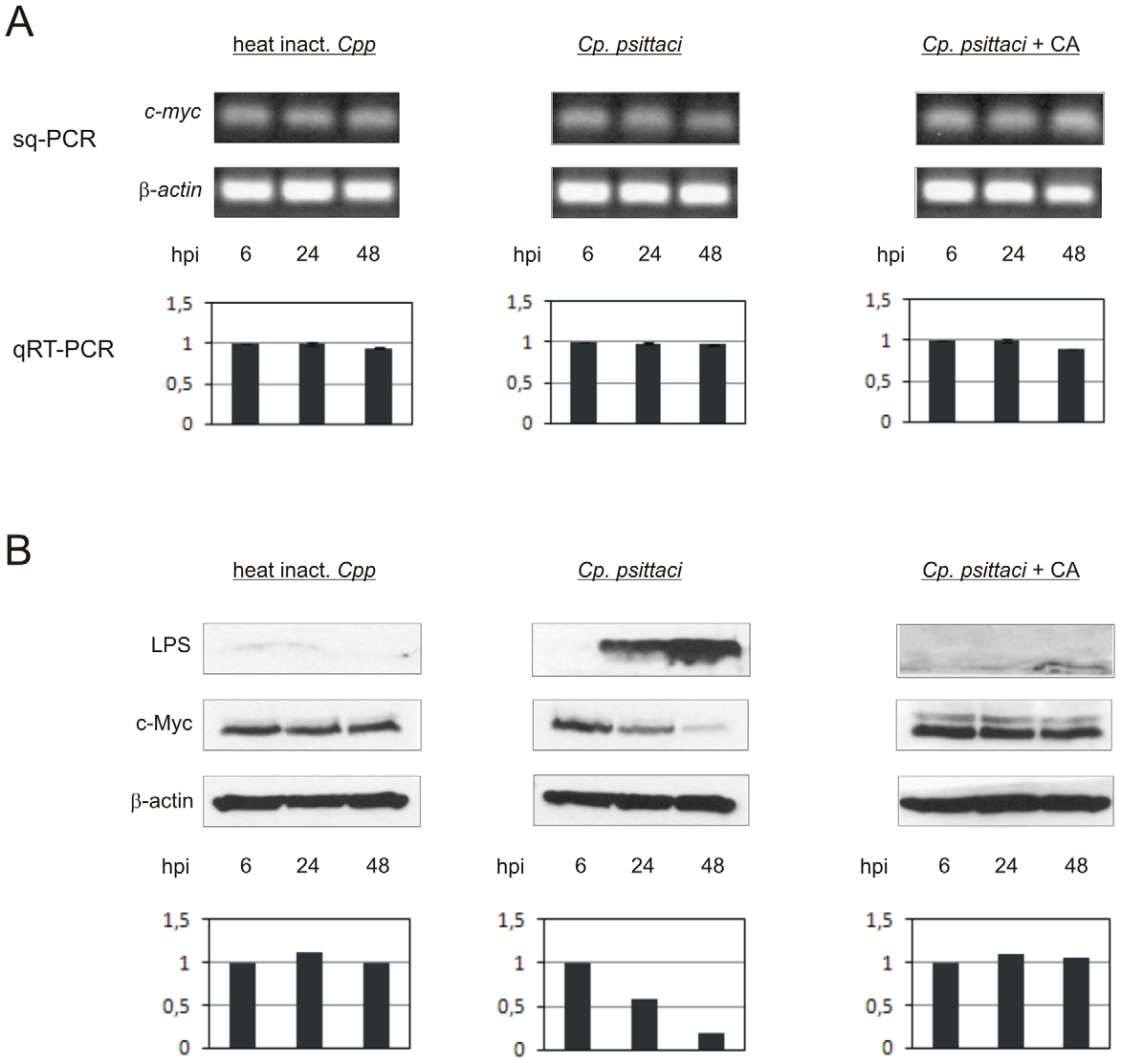
Infection of Hep-2 cells with *Cp. psittaci* leads to a decrease in c-Myc protein concentration. Hep-2 cells were infected with *Cp. psittaci* at a MOI of 3, with heat-inactivated *Cp. psittaci* or chloramphenicol-treated after infection as controls, and at different hours postinfection (hpi) cells were lysed for RNA or protein analysis. (A, above) Semi-quantitative (sq) PCR shows the unchanged expression of *c-myc* mRNA in infected (middle column), infected and chloramphenicol-treated (right) and control (left) Hep-2 cells. *β-actin* served as an internal control gene. (A, below) Quantitative Real-Time (qRT) PCR. *c-myc* levels were standardized on *GAPDH*. Experiments were performed three times in duplicate. (B) Immunoblots of whole cell lysates of *Cp. psittaci* infected (middle column), infected and chloramphenicol-treated (right) and control (left) cells probed with anti-LPS, anti-c-Myc and anti-β-actin antibodies. Bar diagrams below the immunoblots represent a quantification of c-Myc protein related to β-actin protein concentration. Experiments were performed three times.

As it is known that G3BP1 harbors a phosphorylation-dependent RNase activity which specifically cleaves the 3′-untranslated region of human *c-myc* mRNA [18], we wondered whether changes in *c-myc* mRNA and/or protein concentration would be detectable in *Cp. psittaci*-infected Hep-2 cells. For this purpose, Hep-2 cells were infected with *Cp. psittaci* at a MOI of 3, with heat-inactivated *Cp. psittaci*, or chloramphenicol-treated after infection as controls. Total RNA was isolated at different time points of infection and reverse transcribed into cDNA. Expression levels of *c-myc* mRNA and. *β-actin* as a control were estimated by semi-quantitative PCR (Fig. 4A, above) and by quantitative Real-Time PCR using *GAPDH* for standardization (Fig. 4A, below). In both experiments no differences in *c-myc* mRNA levels during the course of infection were detectable in comparison with the control (Fig. 4A). But to our surprise, the c-Myc protein concentration significantly declined in *Cp. psittaci* infected cells with progressive infection under the same experimental conditions (Fig. 4B, middle column). No changes in c-Myc protein concentration were detected in Hep-2 cells treated with heat-inactivated *Cp. psittaci* (Fig. 4B, left column). This inhibitory effect required bacterial protein synthesis as the level of c-Myc protein was nearly unchanged in *Cp. psittaci*-infected cells treated with chloramphenicol (Fig. 4B, right column). Protein levels of c-Myc were standardized to β-actin (Fig. 4B, below).These findings suggest that infection of Hep-2 cells with *Cp. psittaci* leads to a decrease in the c-Myc protein concentration of the host cell.

### The decrease in c-Myc concentration could be ascribed to the interaction between IncA and G3BP1

**Figure 5:**
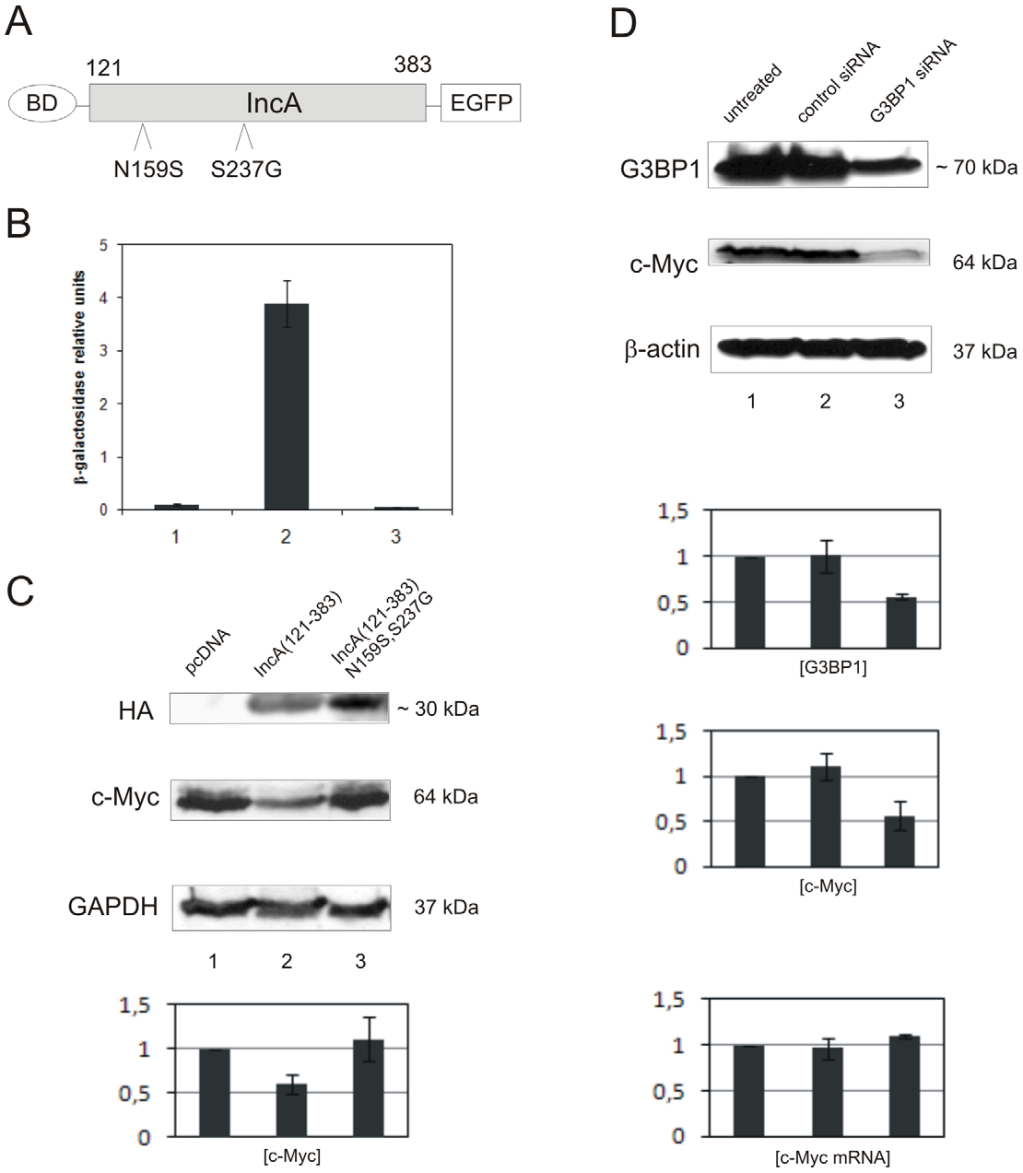
The decrease in c-Myc protein concentration can be ascribed to the interaction between IncA and G3BP1. (A) Schematic structure of the *Cp. psittaci* IncA (aa 121-383) double mutant (N159S; S237G) which is no longer able to interact with G3BP1 in the yeast two-hybrid system. Mutagenesis was performed in *S. cerevisiae* using error-prone PCR. (B) Yeast two-hybrid interaction assay of mutated IncA with G3BP1. Interactions were quantitatively assessed by β-galactosidase liquid culture activity assays. β-galactosidase values represent the mean numbers and standard deviations of three independent experiments. (1) GAL4 BD-IncA (aa 121-383)-EGFP + pGADT7 (negative control), (2) GAL4 BD-IncA (aa 121-383)-EGFP + GAL4 AD-G3BP1 (aa 221-451) (positive control), (3) GAL4 BD-IncA (aa 121-383, N159S; S237G)-EGFP + GAL4 AD-G3BP1 (aa 221-451). (C) Immunoblots of whole HEK293 cell lysates transfected with pcDNA3 (1), pcDNA3-HA-IncA (aa 121-383) (2) or pcDNA3-HA-IncA (aa 121-383, N159S; S237G) (3) and probed with anti-HA, anti-c-Myc and anti-β-actin antibodies. Bar diagram below the immunoblots represent a quantification of c-Myc protein concentration related to β-actin. Experiments were performed in triplicate. (D) Immunoblots of whole Hep-2 cells untreated (1), transfected with non-targeting siRNA (2) or transfected with ON-TARGET G3BP1 siRNA pool and probed with anti-G3BP1, anti-c-Myc and anti-β-actin antibodies. Bar diagrams show knock-down rate of G3BP1 and c-Myc standardized to β-actin and the *c-myc* mRNA concentrations estimated by qRT-PCR. Experiments were performed in triplicate.

To find out whether the decrease in c-Myc protein concentration during *Cp. psittaci* infection of mammalian cells is somehow connected with chlamydial IncA, we transfected HEK293 cells with a mammalian expression vector containing the HA-tagged codon-adapted cytoplasmic domain of IncA (aa 121-383) from *Cp. psittaci*, or the vector only as a control. Overexpression of this IncA fragment in HEK293 cells led to a decrease of c-Myc protein concentration in the same manner as in *Cp. psittaci-*infection of Hep-2 cells (Fig. 5C, lanes 1 and 2). This prompted us to hypothesize that the decrease in c-Myc protein concentration was caused by the recruitment of G3BP1 to the cytoplasmic portion of *Cp. psittaci* IncA. To verify this hypothesis we generated a mutant of IncA (aa 121-383) that was no longer able to interact with G3BP1. Mutagenesis as performed in *S. cerevisiae* using error-prone PCR (see Materials and Methods section) resulted in an IncA (aa 121-383) double mutant (N159S; S237G), which failed to interact with G3BP1 in the yeast two-hybrid system (Fig. 5A and B, column 3). The following overexpression of this mutant in HEK293 cells had no effect on c-Myc protein concentration (Fig. 5C, column 3). Assuming that recruitment of G3BP1 to IncA on the surface of inclusion is responsible for decrease in c-Myc protein concentration, siRNA-mediated knockdown of G3BP1 should have a similar effect. We therefore treated Hep-2 cells with a pool of four siRNAs specific for G3BP1 and as a control with non-targeting siRNAs and analyzed the G3BP1 and c-Myc protein concentrations by Western blot (Fig. 5D). As expected, downregulation of G3BP1 at approximately 50% led to a corresponding downregulation of c-Myc whereas the treatment of cells with control siRNA had no effect (Fig. 5D). As in the case of infection of Hep-2 cells by *Cp. psittaci* the *c-myc* mRNA level remained unchanged. We therefore conclude that the decrease in c-Myc protein concentration resulting from IncA (aa 121-383) in HEK293 cells and most likely also from infection of Hep-2 cells with *Cp. psittaci* can be ascribed to the interaction between IncA and G3BP1.

## Discussion

All *Chlamydiaceae* express a TTS apparatus [8] capable of mediating specific delivery of anti-host effector proteins either into the chlamydial inclusion membrane or into the cytoplasm of target eukaryotic cells [10], [7]. Prominent examples of the first group of anti-host effector proteins are the Inc proteins. IncA was the first member of the Inc protein family to be identified, and it is still the only member of the Inc family for which a function has been proposed, namely involvement in the homotypic fusion of inclusions in *C. trachomatis*. In this process, the already mentioned SNARE-like motif within the cytoplasmic domain of IncA may play a role [5]. Despite the low sequence similarity among IncA proteins of the various *Chlamydiaceae spp.*, conservation of biochemical properties of IncA proteins from *Cp. caviae* and *C. trachomatis* was found [5]. Both proteins self-associate to form multimers. When artificially expressed by the host cell, they localize to the endoplasmatic reticulum and completely inhibit concomitant inclusion development when additionally infected with *Cp. caviae* or *C. trachomatis.*

While the present knowledge on IncA functionality almost entirely refers to the human pathogens *C. trachomatis* and *Cp. pneumoniae*, we focused on the IncA protein of the zoonotic agent *Cp. psittaci* using a yeast two-hybrid screen to search for interacting host proteins that could indicate further functions of this protein. Having identified human G3BP1 as an interaction partner of IncA of *Cp. psittaci* in yeast, we conducted GST pull-down and co-immunoprecipitation experiments that confirmed the interaction between both proteins *in vitro* as well as *in vivo* in HEK293 cells transfected with an IncA-expression construct. Moreover, an accumulation of G3BP1 around the chlamydial inclusion was shown in *Cp. psittaci*-infected Hep-2 cells by means of fluorescence microscopy.

Consequently, the question of functional relevance of the IncA-G3BP1 interaction arose. As shown by Gallouzi *et al.*, G3BP1 is hyperphosphorylated on serine residues and harbors a phosphorylation-dependent RNase activity that specifically cleaves the 3′-untranslated region of human *c-myc* mRNA [18]. Rather unexpectedly, our finding that *c-myc* mRNA concentrations were practically unchanged in the course of *Cp. psittaci* infection of Hep-2 cells was in contrast to the significant decrease in c-Myc protein. Overexpression of IncA in HEK293 cells caused the same effect, whereas an IncA mutant disabled to interact with G3BP1 in yeast did not cause a decrease in c-Myc protein concentration. Moreover, siRNA-mediated knockdown of G3BP1 in Hep-2 cells also downregulated c-Myc. This suggests that the effect could be ascribed to the interaction between IncA and G3BP1. Absence of the possibility to modify *Chlamydia* experimentally precluded us from proving this hypothesis in more detail. We have currently no definitive explanation for a connection between IncA-G3BP1 interaction and c-Myc concentration. An intriguing possibility is that interaction between these proteins disrupts a still unknown c-Myc-stabilizing interaction of G3BP1 with other host proteins thereby rendering c-Myc susceptible to degrading activity. An example for such a protein-stabilizing interaction of G3BP1 is the complex with USP10, a ubiquitin-specific protease, described for human [23] and yeast cells [24]. USP10 removes ubiquitin from proteins to which it is attached, leading to stabilization of the protein. At least for *S. cerevisiae* it was shown that USP10 requires an additional protein, i.e. G3BP1, to form an active de-ubiquitination complex that cleaves ubiquitin from specific substrates [24]. Whether the USP10 – G3BP1 complex is relevant for the stabilization of c-Myc protein in human cells remains to be elucidated.

The c-Myc transcription factor is a powerful regulator of cell growth, proliferation, differentiation and apoptosis [25], [26]. Given its potent effects on cell fate, it is not surprising that cells have employed sophisticated methods for ensuring proper c-Myc expression levels. The expression of c-Myc is regulated at every possible level: transcriptionally (initiation and elongation), post-transcriptionally (mRNA stability and translation) and post-translationally (protein stability) [27]. In 1994, the Eick and Hay laboratories provided evidence that c-Myc deregulation activates the tumor suppressor p53 and triggers apoptosis, a process, highly relevant for chlamydial development [28], [29]. Zindy and colleagues in the Roussel laboratory showed mechanistically that deregulated c-Myc upregulates Arf, which in turn activates p53 to regulate a cohort of target genes involved in apoptosis [30]. Afterwards several other c-Myc-induced pathways that contribute to the apoptotic response were described [31]. Obligate intracellular *Chlamydiaceae* depend on an intact host cell for replication and production of infective progeny. Therefore, they possess diverse strategies to prevent apoptotic cell death in the host [32]. One of these is the proteasomal degradation of pro-apoptotic BH3-only proteins by the chlamydial protease/proteasome-like activity factor (CPAF) [33]. Another successful mechanism is the upregulation of anti-apoptotic Bcl-2 like proteins such as Mcl-1 [34]. We suggest here another possible strategy employed by *Cp. psittaci* to overcome host cell apoptotic mechanisms, i.e. the inhibition of c-Myc-driven apoptotic events by lowering c-Myc protein concentration.

In addition to apoptosis regulation, another aspect of c-Myc effects on cell fate is highly relevant to chlamydial development, i.e. the regulation of cell cycle and, therefore, proliferation. c-Myc enables cell growth by providing the cell with an abundant supply of several classes of basic building blocks, as well as increasing cell metabolism and protein synthesis. Several c-Myc target genes are thought to have a role in this activity, including those associated with cellular metabolism, ribosomal and mitochondrial biogenesis, as well as protein and nucleic acid synthesis [27]. Furthermore, expression profiling data indicate that c-Myc has the capacity to both stimulate cell growth and abrogate cell cycle inhibitors, a powerful combination of functions that, when deregulated, may drive the limitless replicative potential characteristic of nearly all tumors [26]. Regarding *Chlamydiaceae*, it is widely accepted that they develop predominantely within terminally differentiated epithelial cells of mammalian mucosae and not within actively dividing cells [35]. Furthermore, studies on *C. trachomatis* showed that host cells infected with *C. trachomatis* undergo a lower rate of cell division than their uninfected counterparts [36], and that *C. trachomatis* infection inhibits host cell cytogenesis [37] and induces cleavage of the mitotic cyclin B1 [38]. We propose that the downregulation of c-Myc protein concentration during infection of Hep-2 cells with *Cp. psittaci* is an alternative way to reduce host cell proliferation and so to establish optimal conditions for development of this obligate intracellular microorganism within the host cell.

## Materials and Methods

### Cell culture and organisms

Hep-2 cells (ATCC-CCL23) were grown in DMEM medium with 4.5 g/l glucose (PAA) supplemented with 10% heat-inactivated fetal calf serum (FCS) and maintained at 37°C in an atmosphere of 5% CO_2_. Sub-confluent monolayers were inoculated with *Cp. psittaci* DC15 (bovine isolate, genotype A [39]) or heat-inactivated DC15 at a multiplicity of infection (MOI) of 3. After centrifugation at 2.000 rpm and 37°C for one hour they were maintained as described above. For inhibition of chlamydial protein synthesis cells were treated with 200 µg/ml chloramphenicol. HEK293 cells (ATCC-CRL1573) were cultivated in RPMI medium with L-glutamine (PAA) supplemented with 10% heat-inactivated FCS under mentioned conditions.

### Mammalian expression constructs and transfections

To obtain an optimal expression of IncA in human cells full-length DNA sequence of IncA of *Cp. psittaci* was optimized for human codon usage (sequence available on demand), *in vitro* synthesized (GenScript) and cloned as an EcoRI-NotI-fragment into pcDNA3.1/*myc*-His A (Invitrogen). To express HA-tagged codon adapted cytoplasmic domain of IncA (aa 121-383) from *Cp. psittaci* in mammalian cells the corresponding DNA fragment was amplified by PCR using the following primers: forward-5′- GCGAATTCACCATGTACCCATACGATGTTCCAGATTACGCTACCCCCAGCCAGGTGGCCCGG-3′, reverse-5′-CGCCTCGAGTTACTGCTCGTAGATGGGGAAGCCCT-3′ and the full-length pcDNA3.1/*myc*-His A-construct (see above) or the mutated construct (see below) as template. EcoRI-XhoI-digested PCR fragments were transferred into pcDNA3 (Invitrogen). HEK293 cells were transfected with FuGENE® HD transfection reagent according to the manufacturer's instructions (Roche) and incubated for 24 hours.

For siRNA experiments Hep-2 cells were transfected with ON-TARGET *plus* Non-Targeting siRNA #1 or ON-TARGET *plus* SMART pool human G3BP1 using DharmaFECT 1 transfection reagent as described by the manufacturer (Thermo Scientific Dharmacon®), maintained for 72 hours and lysed as mentioned below.

### Co-immunoprecipitation

HEK293 cells were transfected with pcDNA3.1/*myc-*His full-length codon-adapted IncA expression construct and maintained for 24 hours. Co-immunoprecipitation was performed with µMACS Protein G microbeads as described by the manufacturer (Miltenyi Biotec). Transfected HEK293 cells were washed twice with cold PBS, harvested and resuspended in low-salt lysis buffer. Cell lysate was incubated with monoclonal mouse anti-His antibody (Roche) and µMACS Protein G microbeads on ice. After 45 minutes magnetic separation using microcolumns and the µMACS separator was performed. Samples were extensively washed and co-immunoprecipitated proteins were eluted from the column with SDS gel loading buffer.

### Immunoblotting

For immunoblotting of co-immunoprecipitated proteins, samples were loaded on a 10% SDS-PAGE and transferred to a nitrocellulose membrane (Whatman). Membranes were blocked with 5% skim milk in TBST for 1 h and incubated overnight at 4°C with mouse anti-His (Roche) or mouse anti-G3BP1 (Santa Cruz Biotechnology) primary antibodies. After washing three times with TBST membranes were incubated with horseradish peroxidase-conjugated goat anti-mouse secondary antibody (Dianova). For detection of protein bands ECL Plus Western Blotting Detection Reagent (GE Healthcare) was used as recommended. For Western blot analyses of infection experiments, Hep-2 cells were collected at different time points, washed twice with cold PBS and lysed using RIPA-buffer (150 mM NaCl, 65 mM Tris-HCl pH 7.5, 1% sodium deoxycholate, 1% Triton-X 100, 0.1% SDS). For detection of protein bands mouse anti-c-Myc (Santa Cruz Biotechnology), mouse anti-β-actin (Sigma Aldrich) and mouse anti-chlamydial LPS (AbD Serotec) primary antibodies and goat anti-mouse secondary antibody (Dianova) were used. Hep-2 cells treated with siRNA and HEK293 cells transfected with HA-tagged IncA expression vectors were treated equally. Detection occurred with monoclonal mouse anti-HA, mouse anti-G3BP1 and mouse anti-c-Myc (all obtained from Santa Cruz Biotechnology) primary antibodies and secondary anti-mouse antibody (Dianova).

### Immunofluorescence and confocal microscopy

Hep-2 cells grown on coverslips were transfected with DsRED-G3BP1 expression construct (obtained from Zhi-Min Yuan, Harvard School of Public Health, Boston, USA) or DsRed vector. After 24 hours cells were infected with *Cp. psittaci* DC15 at a MOI of 3 and incubated for additional 48 hours. Infected and uninfected Hep-2 cells were washed with PBS and fixed with 3,8% paraformaldehyde. For neutralization cells were washed twice with 0,1 M glycin/PBS followed by permeabilization in 0,5% NP-40/PBS for 10 min. Then cells were blocked with PBS containing 0,1% NP-40 and 5% FCS overnight at 4°C. Immunostaining of endogenous G3BP1 occurred with polyclonal rabbit anti-G3BP1 antibody (Abcam), inclusions were visualized using mouse anti-chlamydial LPS antibody (AbD Serotec) by incubation for 1, 5 h at room temperature. After washing four times with 0,1% NP-40/PBS secondary FITC-conjugated goat anti-rabbit (Dianova) and RRX-conjugated goat anti-mouse (Dianova) were incubated for 1 h. All samples were additionally stained with 0,1 µg/ml DAPI, extensively washed with 0,1% NP-40/PBS, PBS and A. bidest. and mounted with MountFluor (Pro Taqs). Images of infected and immunostained Hep-2 cells were collected with a BX51 fluorescence microscope (Olympus) or an Axiovert 200 M/LSM 510 META laser scanning confocal microscope (Carl Zeiss, Jena) according to [40].

### RNA isolation, semi-quantitative PCR and qRT-PCR

Hep-2 cells were infected as described above and collected at different time points. For RNA purification RNeasy Mini Kit and QIAshredder (Qiagen) were used according to manufacturer's instructions. For DNA digestion samples were treated with RQ1 RNase-free DNase (Promega) and purified with RNA Clean and Concentrator (Zymo Research) according to usage informations. Reverse transcription was done using M-MLV Reverse Transcriptase RNase H Minus (Promega), 0, 5 µg RNA, 1 µg oligo(dT)_12-18_ (Roth), 10 mM of each dNTP, 5 µl of 5× reaction buffer, 40 U of RiboLock^TM^ RNase inhibitor (Fermentas) and 100 U M-MLV RT (H-) in a total volume of 25 µl according to manufacturer's instructions. With all RNA samples control reactions without reverse transcriptase during production of the cDNA which was subsequently used in the PCR were performed. For semi-quantitative PCR with 1 U *Pwo*-DNA-Polymerase (Peqlab), 1 µl of cDNA sample, 10 mM of each dNTP, 5 µl of 10× *Pwo*-reaction buffer and 10 µM of according primers were used in a total volume of 50 µl. Primer set: *c-myc* for: 5′-GCCAGAGGAGGAACGAGCT-3′ and *c-myc* rev: 5′-GGGCCTTTTCATTGTTTTCCA-3′; *β-actin* for: 5′-CTGGAACGGTGAAGGTGACA-3′ and *β-actin* rev: 5′-AAGGGACTTCCTGTAACAATGCA-3′. The following temperature-time profile was used: initial denaturation at 95°C for 5 min, 25 cycles of 95°C for 1 min, 55°C for 45 sec and 72°C for 45 sec, and a final elongation step at 72°C for 2 min. PCR products were electrophoresed on an 1% agarose gel containing ethidium bromide and visualized on an UV transilluminator.

For quantitative Real-Time PCR cDNA samples were diluted 1∶10 in RNase-free water. 2 µl of each sample, 0,2 µM of primers, *c-myc* for and *c-myc* rev (see above) or *GAPDH* for: 5′-TGCACCACCAACTGCTTAGC-3′ and *GAPDH* rev: 5′-GGCATGGACTGTGGTCATGAG-3′, and SsoFast^TM^ EvaGreen® supermix with Low ROX (BIO-RAD) in a total volume of 20 µl were used. PCR was performed in StepONE Real-Time PCR-system: 95°C for 2 min, 40 cycles of 95°C for 5 sec and 62°C for 20 sec, and analyzed with StepONE software v2.0 (Applied Biosystems).

### Yeast two-hybrid assay

The full-length IncA of *Cp. psittaci* DC15 was PCR amplified using chromosomal DNA of this species as template and the following primer sets: IncAPsi1: 5′-GCGAATTCATGACATCACCAGTAGAATCT-3′ and IncAPsi2: 5′-GCGGATCCTTACTGTTCATATATTGGGAA-3′ (aa 1-383). The cytoplasmic domain of IncA protein was PCR amplified in an analogous manner using the following primer set: IncAPsi4: 5′-GCGAATTCACTCCCTCTCAAGTGGCACGC-3′ (aa 121-383) and IncAPsi2. PCR fragments were digested with EcoRI-BamHI and transferred into the GAL4 BD destination vector pGBKT7 (CLONTECH). The C-terminal deletion fragment of G3BP1 was PCR amplified using the following primer set: G3BP1d: 5′-GCGAATTCGATGATTCTGAGCCTGTTCAG-3′ and G3BP1b: 5′-GCCGTCGACTCACTGCCGTGGCGCAAGCC-3′and DsRed-G3BP1 vector (kindly provided by Zhi-Min Yuan, Harvard School of Public Health, Boston, USA) as a template. PCR fragment was digested with EcoRI-SalI and transferred into the GAL4 AD destination vector pGADT7 (CLONTECH). *S. cerevisiae* Y190 (CLONTECH) expressing pGBKT7-IncA (aa 121-383) was mated for 6 hours with *S. cerevisiae* Y187 expressing the HeLa cDNA MATCHMAKER 3 library in the activation domain (AD) vector pGADT7-Rec (CLONTECH). Interacting clones were first selected by growth on vector-selective medium also lacking histidine but containing 5 mM 3-amino-1,2,4-triazole (3-AT). Emerging colonies were checked for activity of the second reporter gene *lacZ* by performing the colony-lift filter assay using X-Gal (5-Bromo-4-chloro-3-indolyl-α-D-galactopyranoside) as a substrate. HeLa cDNA-expressing plasmids were recovered from these strains, propagated in *E. coli* and sequenced to identify coding sequences. For quantitative two-hybrid assays *S. cerevisiae* Y190 was transformed with corresponding pGBKT7- and pGADT7-constructs and β-galactosidase liquid culture assays, using o-nitrophenyl β-D-galactopyranoside (ONPG) as substrate were performed according to the manufacturer's instructions (CLONTECH) [41]. The β-galactosidase values represent the mean numbers and standard deviations of three independent experiments.

### Mutagenesis screen procedure

Construction of a mutated cytoplasmic domain of *Cp. psittaci* IncA disabled to interact with G3BP1 was performed in *S. cerevisiae* using error-prone PCR as already described [42]. Briefly, codon adapted cytoplasmic domain of IncA (aa 121-383) from *Cp. psittaci* was amplified by PCR analogous to the mammalian construct (see above) and cloned as a NdeI-EcoRI-fragment into pGBKT7 in frame with the enhanced green flourescence protein (EGFP) from pEGFP-C1 vector (CLONTECH) resulting in a GAL4 DB-IncA-EGFP fusion protein. The EGFP protein allowed to exclude frame shift mutations in the mutated IncA domain constructs. PCR primers (T7SEQ: 5′-GTAATACGACTCACTATAGGGCGA-3′ and GFPrev: 5′-GCTGAACTTGTGGCCGTTTAC-3′) flanking the IncA domain were used to amplify this region using error-prone PCR conditions as described [42] but utilizing SAWADY Taq-DNA-Polymerase (PEQLAB). The liniarized GAL4 DB-EGFP plasmid and the randomized PCR fragment were then co-transformed into yeast strain Y190 expressing the GAL4 AD-G3BP1 library plasmid (Fig. 1B). The high efficiency of homologous recombination in yeast, between the linearized plasmid and the PCR fragment, enabled expression of randomized cytoplasmic IncA domain chimeric proteins within yeast cells, permitting detection of those not binding to G3BP1 but still expressing EGFP via flourescence microscopy and colony-lift filter assay as described above.

### GST pull-down

To produce GST-tagged IncA protein fragments, full-length and C-terminal cytoplasmic fragments from IncA of *Cp. psittaci* DC15 were transferred from corresponding pGBKT7-constructs (see Yeast two-hybrid assay section) into expression vector pGEX-4T-1 (GE Healthcare) encoding GST. To obtain GST-tagged full-length G3BP1 protein (aa 1-466) the corresponding DNA fragment was amplified by PCR using the following primers: forward-5′-GCGAATTCATGGTGATGGAGAAGCCTTAGT-3′, reverse-5′-GCCGTCGACTCACTGCCGTGGCGCAAGCC and DsRED-G3BP1 vector as template. EcoRI-SalI-digested PCR fragment was transferred into pGEX-4T-1. Plasmids containing sequences encoding GST, *Cp. psittaci* DC15 GST-IncA (aa 1-383), GST-IncA (aa 120-383) and GST-G3BP1(aa 1-466) were transformed into *E. coli* strain BL21-CodonPlus-RIL (Stratagene). 13 ml portions of overnight cultures were filled up to 20 ml with LB medium containing 100 µg of ampicillin/ml and fusion derivates were induced with 1 mM isopropyl β-D-thiogalactoside (IPTG) for induction of protein expression. After 1,5 h incubation cells were collected by centrifugation, resuspended in 1 ml PBS containing 1 mM PMSF and 10 mM DTT, and then sonicated. After sonification 1% Triton X-100 was added and samples were incubated for 30 min at 4°C. Then the lysates were cleared by centrifugation and the supernatants were subjected to Glutathione Sepharose affinity chromatography for purification of the GST fusion proteins as described by the manufacturer (GE Healthcare). [^35^S]-methionine-labeled G3BP1 full-length and IncA cytoplasmic domain proteins were synthesized *in vitro* by coupled T7 RNA polymerase-mediated transcription and translation (IVT) in a reticulocyte lysate system as described by the manufacturer (Promega). Glutathione Sepharose beads (50 µl) liganded by either GST-tagged protein fragments or GST alone were washed with HB-buffer (20 mM HEPES, 100 mM KCl, 5 mM MgCl_2_, 0,5% Igepal-CA630, 0.5 mM dithiothreitol, pH 7.4) and then resuspended in 100 µl HB-buffer containing 0.5% Igepal CA630 and 10 µl [^35^S]-methionine-labeled protein. After incubation at 4°C by constantly rotating for 2 hours, the beads were washed with HB-buffer extensively. Proteins were eluted from the beads using SDS sample buffer. After separation by 10% SDS-PAGE, G3BP1 and IncA cytoplasmic domain were revealed by exposure on KODAK X-OMAT AR film (Kodak). Analogous GST pull-down experiments were performed with Hep-2 cell lysates instead of [^35^S]-methionine-labeled G3BP1. The associated proteins were pelleted by centrifugation, washed and analyzed for full-length G3BP1 by Western blot analysis as described in the co-immunoprecipitation and Immunoblotting sections.
